# The Effect of Health Literacy Intervention on Patients with Diabetes: A Systematic Review and Meta-Analysis

**DOI:** 10.3390/ijerph192013078

**Published:** 2022-10-12

**Authors:** Xue Ran, Yalan Chen, Kui Jiang, Yaqin Shi

**Affiliations:** Department of Medical Informatics, Medical School, Nantong University, Nantong 226001, China

**Keywords:** health literacy, diabetes, meta-analysis, systematic review, intervention

## Abstract

Relevant studies published between January 2010 and June 2021 were identified through relevant databases, including the Science Citation Index Expanded (SCIE) database of Web of Science, PubMed, and Embase, in order to assess the effect of health literacy (HL) intervention on patients with diabetes. A total of 21 articles were eligible. The results showed that: (1) this review involved different HL assessment tools, most of which were self-designed scales and assessment tools focused on measuring functional HL. (2) The differences in glycosylated hemoglobin (HbA1c) (weighted mean difference [WMD] = −0.78, 95% confidence interval [CI]: −0.94, −0.62) and medication adherence (standardized mean difference [SMD] = 1.85, 95% CI: 0.19, 3.52) between the HL intervention group and the usual care group were statistically significant. There was no significant improvement in systolic blood pressure (SMD = −0.05, 95% CI: −0.34, 0.25). Furthermore, this review reported that self-efficacy (SMD = 0.85, 95% CI: 0.65, 1.04) was increased, and the level of HL was improved. In the assessments of risk of bias, 90% of the studies were classified as medium. The quality of the evidence of medication adherence was very low, and the reliability of the conclusions was not enough to confirm the effect of HL.

## 1. Introduction

Diabetes is one of the most common chronic diseases. Poor control of intermediate risk factors (e.g., blood pressure and glycemic control) and genetic susceptibility are associated with an increased risk of adverse outcomes in patients with diabetes in the long-term treatment process. Non-clinical factors such as socioeconomic and psychosocial characteristics also play a key role in determining the risk for a range of adverse health outcomes, including heart attacks, strokes, amputations, blindness, and end-stage renal disease [1].

Health literacy (HL) is an important non-clinical factor to reduce the risk of adverse outcomes of diabetes. So far, the definition of HL has not reached a unified standard. Most definitions encompass the two interacting parts: the patient’s personal skills and their environment around them, such as the healthcare service and the environment where they live. Currently, the generally accepted definition of HL given by the National Library of Medicine (NLM) is defined as “the degree to which individuals have the capacity to obtain, process, and understand basic health information and services needed to make appropriate health decisions [2,3,4]”. A health literacy friendly organization can improve the patient’s HL skills. Published findings suggest that the importance of HL is increasingly recognized by healthcare workers, and there is a call action to improve HL, particularly among people with chronic diseases (e.g., diabetes) and other vulnerable groups (e.g., the elderly) [5]. Therefore, in the diabetes health education process, it is necessary to develop effective HL interventions to disseminate understandable health information to patients with diabetes, which is conducive to the maintenance of their own health.

Many recent studies have proposed various interventions to improve the health of patients with diabetes and limited HL. Wei et al. [6] found that HL intervention (the Partnership to Improve Diabetes Education Toolkit (PRIDE) and a Clear Health Communication Curriculum) can effectively improve glycemic control in Chinese people with type 2 diabetes mellitus. The Theory of Planned Behavior (TBP) based educational intervention by Zeidi et al. [3] can significantly increase the HL of people with type 2 diabetes mellitus. In addition, it can promote the improvement and maintenance of self-care behaviors (e.g., blood glucose self-monitoring, healthy diet, and foot care). Moura et al. [7] conducted educational interventions (including three meetings to train patients with type 2 diabetes for the adoption of appropriate behaviors considering their condition) through three weekly meetings, with an average duration of approximately 60 min, indicating a positive effect on self-care and functional HL. Sugita et al. [8] intervened by sending HL-related messages to the experimental group and found that there were no significant differences between the two groups in medication adherence, level of HL, glycosylated hemoglobin (HbA1c), and other results (*p* > 0.05). Although many studies have examined the effect of HL intervention on patients with diabetes, there is little consensus. Therefore, our objective was to perform a systematic review and meta-analysis of published studies on HL intervention for people with diabetes to determine their effectiveness in implementing effective HL intervention in people with diabetes.

## 2. Methods

We adhered to the Preferred Reporting Items for Systematic Reviews and Meta-Analyses (PRISMA) statement, and the PRISMA checklist for this study is provided in Table S1. In addition, the protocol has been registered in the PROSPERO registry (CRD42021284053).

### 2.1. Data Source and Searches

A literature search was conducted in the Science Citation Index Expanded (SCIE) of Web of Science, PubMed and Embase for relevant intervention studies published between January 2010 and June 2021. A wide range of text words and indexed terms related to “diabetes mellitus” and “HL” were comprehensively searched. The detailed search strategy is presented in Table S2.

### 2.2. Eligibility Criteria

The inclusion criteria were: (1) types of studies: randomized controlled trials (RCTs), controlled before-and-after trials (CBAs), quasi-experimental studies (QEs); (2) study participants: people with diabetes; (3) types of interventions: interventions related to HL, form, type, and duration were not limited; (4) outcomes: HL level, HbA1c, systolic blood pressure (SBP), self-efficacy, and medication adherence (limited by of outcome indicators of included studies).

The exclusion criteria were: (1) study participants with other serious diseases, such as cognitive impairment; (2) conference reports and review articles, among others; (3) studies with insufficient data (e.g., protocols, conference proceedings, or abstracts) and without the author’s response to our request; (4) duplicated reports.

### 2.3. Data Extraction

Two reviewers independently screened relevant studies and then extracted relevant data according to pre-specified inclusion and exclusion criteria. If there were disagreements in cross-validation, the inclusion was determined by consensus after discussion or by a third reviewer. Using a pre-designed data extraction form, the main contents extracted from each study included basic information of literature and primary outcomes.

### 2.4. Quality Assessment of Studies

Two reviewers assessed the quality of included studies separately using the Cochrane Effective Practice and Organization of Care Review Group (EPOC) [9] standards. When discrepancies occurred regarding a certain item, a third reviewer was consulted. The criteria were: (1) random sequence generation; (2) allocation concealment; (3) baseline measurement criteria; (4) baseline characteristics before intervention; (5) data comprehensiveness; (6) blind implementation of outcome measurement; (7) protection against contamination; (8) selective reporting; and (9) other risks of bias. The overall score of risk of bias for the included studies was determined by nine aspects of the tool and summarized into three ratings: “Yes” (low-bias risk), “Unclear”, or “No” (high-bias risk).

### 2.5. Data Synthesis and Analysis

The results were summarized qualitatively and quantitatively. A meta-analysis was conducted with Stata 16.0 software. For all analyses, statistical significance was accepted at *p* < 0.05. Heterogeneity between studies was analyzed by $x^{2}$ test, and *p* < 0.1 indicated heterogeneity. The weighted mean difference (WMD) and the standardized mean difference (SMD) were applied for continuous variables. If there were multiple follow-up time points in the study, the data collected at the last follow-up were extracted. If there were multiple intervention groups in the study, data from the experimental group were selected only involving an HL intervention. RCTs were preferred for meta-analysis. If there were no available data (e.g., there was no difference in the study results, data did not use the mean and standard deviation, or it was difficult to convert into data of interest), CBAs were chosen. Sensitivity analysis was performed by a statistical transformation of the model.

### 2.6. GRADE Summary of Findings

The certainty of the evidence for each outcome was assessed independently by two reviewers using the Grading of Recommendations, Assessment, Development and Evaluation (GRADE) [10] approach. The assessment included the following five aspects: (1) risk of bias; (2) inconsistency; (3) indirectness; (4) imprecision; (5) other considerations, namely, publication bias (RCTs), large effect, plausible confounding, and dose response gradient (observational study). A summary of findings table was generated using GRADEpro GDT (Guideline Development Tool) software for the outcomes. The quality of evidence across each outcome was eventually classified into four levels (high, moderate, low and very low).

## 3. Results

### 3.1. Study Selection

**Figure 1:**
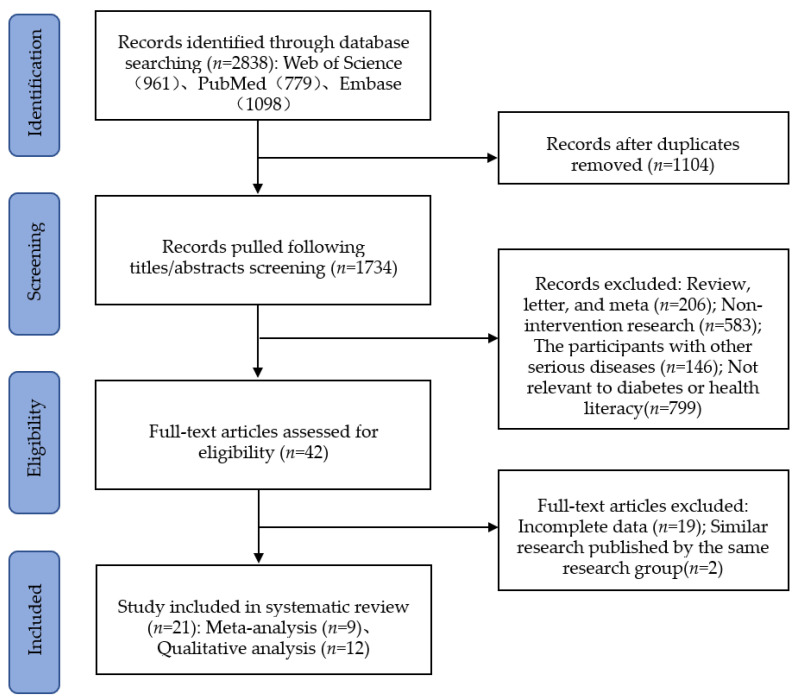
Flow diagram of study selection.

The initial search identified 2838 articles, of which 1734 were reviewed based on titles and abstracts after removing duplicates. Further, 1692 articles were excluded according to review criteria. Of the remaining 42 full-text studies retrieved, 21 were excluded because they did not present data of interest or were repeated articles. Ultimately, 21 articles [2,3,6,7,8,11,12,13,14,15,16,17,18,19,20,21,22,23,24,25,26] were included for systematic review. Because the data homogeneity of 12 studies was not high, these were included in a qualitative analysis, and a meta-analysis was conducted for the other nine articles. The results of the literature search and study selection are shown in Figure 1.

### 3.2. Characteristics of the Included Studies

The basic characteristics of 21 studies included in the systematic review are summarized in Table S3, including author information, geographical area, publication year, study type, sample size, intervention, and other information. A total of 3402 participants were included in the review. Eleven of the studies were RCTs, six were CBAs, and the remaining studies were QEs. The duration of the intervention ranged from three weeks to two years. More than a third of the studies were conducted in the United States, with three in Iran, two in Canada, and one in Mexico, Germany, Japan, China, Taiwan, Brazil, Thailand, and England. The study interventions were mainly centered on HL and behavior-based education.

### 3.3. Results of the Risk of Bias Assessment

Eleven studies were RCTs, of which 45% did not specify the randomization method. Only two studies [11,17] achieved the double-blind of the outcome measurer and the study participant. None of the included studies indicated whether measures were taken to prevent data contamination. Similar baseline characteristics and incomplete outcome data were generally low risk for all studies, except one [26]. Overall, two studies were rated as low (high risk of bias), 19 studies had an overall quality evaluation of medium (Table S4).

### 3.4. Systematic Analysis of HL Assessment Tools

The study involved different tools in assessing HL, with the Test of Functional Health Literacy in Adults (TOFHLA) [3,16,17,24] being the most widely used. The tools of assessment of HL involved in our study focused on the ability to understand the reading of patients with diabetes, namely functional HL, such as TOFHLA, Newest Vital Sign (NVS) [13,16,25], Rapid Estimate of Adult Literacy in Medicine (REALM) [16,24], and Medical Term Recognition Test (METER) [13]. The assessment tools involving the three types of HL (e.g., functional HL, communicative HL and critical HL) include FCCHL (Functional, Communicative, and Critical Health Literacy Scale) [8], the Iranian Health Literacy Questionnaire (IHLQ) [19] and the Health Literacy Questionnaire (HLQ) [12]. The detailed evaluation contents of the scales are shown in Table S5. The items of the HL assessment tool are generally scored according to the dichotomy or the 4–5 point Likert scale. Cronbach’s alpha coefficient is usually used to assess the reliability of the scales.

The assessment tools all consider the improvement of scale score as an effective indicator. In all, 14 studies reported the effect of HL intervention on the HL level of patients with diabetes, of which 11 studies [3,7,13,14,16,17,18,19,22,24,25] showed that the HL level of participants improved after the educational intervention. Three studies [8,12,20] found that there were no statistically significant differences in HL scores.

### 3.5. Meta-Analysis of Clinical Outcome

#### 3.5.1. HbA1c

**Figure 2:**
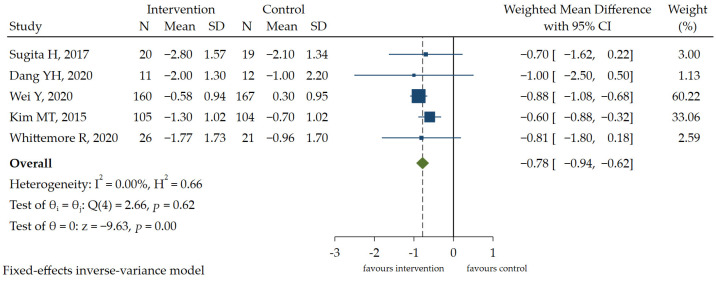
The forest plot of the effect of HL intervention on HbA1c [6,8,11,15,21].

Glucose indicators were reported in 47% of the studies, involving fasting blood glucose, namely FBG, and HbA1c. The information in the literature included in the study related to data on other glucose indicators (e.g., 2 h postprandial blood glucose [2hPBG]) was limited, which complicated the meta-analysis. Only one study [25] involved FBG, and 2hFBG was not mentioned in the included studies. Five RCT studies [6,8,11,15,21] provided enough data to conduct a meta-analysis of HbA1c. The pooled results showed that there is a small but statistically significant difference in the outcome between the experimental group and control group [WMD = −0.78, 95% CI (−0.94, −0.62), *p* < 0.05] (Figure 2), favoring the experimental group. A GRADE analysis indicated that the quality of the evidence supporting this outcome was moderate due to imprecision (Table S6).

#### 3.5.2. SBP

**Figure 3:**
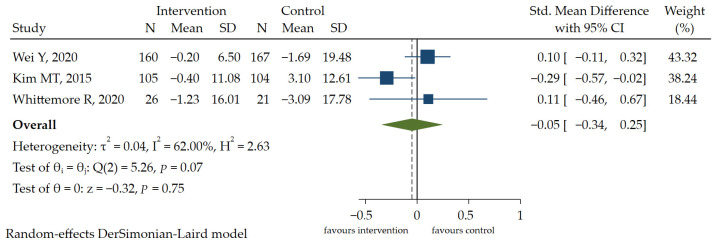
The forest plot of the effect of HL intervention on SBP [6,11,21].

Three RCT studies [6,11,21] reported the effects of interventions on SBP. The results of the meta-analysis showed that there was no significant difference in SBP between the two groups [SMD = −0.05, 95% CI (−0.34, 0.25), *p* > 0.05] (Figure 3). A GRADE analysis indicated that the quality of evidence supporting this outcome was moderate due to imprecision (Table S6). As there were no differences in the results of diastolic blood pressure (DBP) and a consistent conclusion was drawn, DBP was not included in this review for quantitative analysis. In general, HL education (e.g., literacy-numeracy-sensitive diabetes education materials, health communication curriculum) does not have a positive effect on blood pressure control in people with diabetes.

### 3.6. Meta-Analysis of Self-Reported Outcome

#### 3.6.1. Self-Efficacy

**Figure 4:**
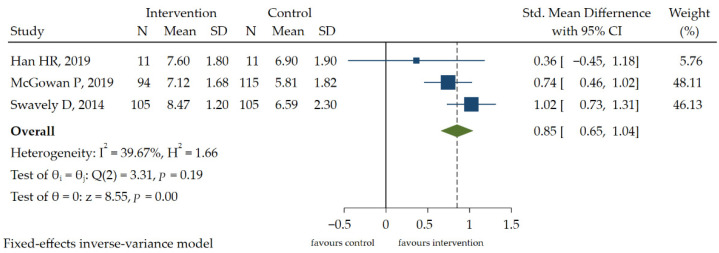
The forest plot of the effect of HL intervention on self-efficacy [20,23,25].

Because the data homogeneity of two RCT studies [8,11] was not high, a meta-analysis was performed on the other three CBA studies [20,23,25]. Three studies used different measurement tools to measure self-efficacy, namely the Stanford Chronic Self-Efficacy Scale [23,25] and the Self-Efficacy Scale [20]. Meta-analysis of interventions that aimed to change self-efficacy showed that the score of the self-efficacy was improved, and the difference was statistically significant [SMD = 0.85, 95% CI (0.65, 1.04), *p* < 0.05] (Figure 4). According to the GRADE approach, this outcome was initially rated low and was ultimately rated very low due to the risk of bias (Table S6).

#### 3.6.2. Medication Adherence

**Figure 5:**
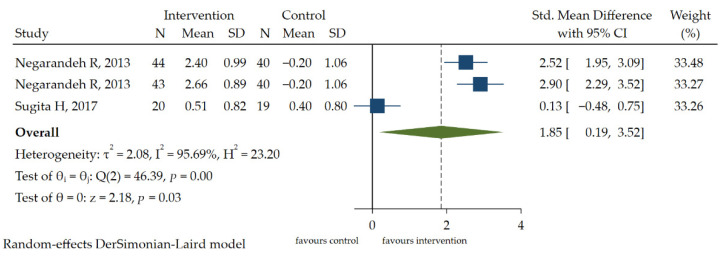
The forest plot of the effect of HL intervention on medication adherence [2,8].

The Morisky Medication Adherence Scale (MMAS-8-Item) [2,8,26] is mostly used in medication adherence assessments, often in people with low HL. Two RCT studies [2,8] reported the effects of interventions on medication adherence. According to the data heterogeneity ($I^{2}$ > 50%, *p* < 0.1), the random effect model was used to analyze the improvement of medication adherence [SMD = 1.85, 95% CI (0.19, 3.52), *p* < 0.05] (Figure 5). A GRADE approach indicated that the quality of evidence supporting this outcome was low due to inconsistency and imprecision (Table S6).

### 3.7. Sensitivity Analysis and Publication Bias

To ensure the stability of the conclusions of the meta-analysis, different statistical models were re-selected for each indicator. It was found that the combined results did not change and stability was good (Table S7). Because fewer than 10 indicators were included in the meta-analysis, publication bias analysis was not conducted [27,28,29].

## 4. Discussion

This systematic review and meta-analysis included 21 pieces of literature, systematically analyzed the geographical distribution of the study and the assessment tools for HL, and summarized the effect of HL interventions on people with diabetes.

More than a third of the studies were conducted in the United States, with two in Korean-American [16,21], two in African-American [23,25] populations, and others in rural and suburban populations of the United States. The adverse effects of low HL are particularly evident among immigrants to the United States who are born in countries where English is not spoken. In areas with limited economic development, it is also difficult to obtain relevant health information. In developing countries such as Iran, China, Brazil and Mexico, there are considerable differences in development among regions, which seriously affect participants’ HL.

HL is generally divided into functional HL, communication HL and critical HL [4]. Some studies pay more attention to a certain type of HL, and the assessment of HL is not comprehensive enough. For example, TOFHLA only measures the level of functional HL, that is, it measures participants’ ability to read and understand information. However, FCCHL can be regarded as the most useful and comprehensive tool for assessing HL. When assessing the HL level of a specific group, it is more effective to use the HL assessment tool specially developed for this group. However, in this study, not all included scales are specifically used to measure the HL level of people with diabetes. Such diabetic-nonspecific scales have difficulty reflecting the characteristics of people with diabetes.

The assessment tool considers the improvement of the scale score as an effective indicator, and higher scores indicate a higher HL level. Overall, HL intervention positively impacts the HL level of patients with diabetes, and the HL score has increased. However, some research results showed that there was no statistical significance in the change in patient health literacy level after intervention. This may be related to the fact that participants had a certain level of HL before being included in the intervention study, suggesting that ceiling effects might have occurred, making the detection of small changes more difficult [12]. In addition, the short intervention time also affects researchers’ observation of the HL level of participants [8,12]. Intervention studies that successfully addressed health literacy often had longer durations, typically lasting nine months to two years [30,31]. Finally, the small study sample size of the included studies may also account for the lack of change in HL [8,12,20].

The results of the meta-analysis showed that health education focusing on HL helped control HbA1c. The reason may be that blood glucose control occupies an important position in the health management of diabetes and has a preference in the design of intervention content. Most interventions focus on blood glucose management. In addition, improvement in self-efficacy and medication adherence may be an intermediary factor in controlling HbA1c. Since DBP cannot be quantitatively analyzed, these data were not included for meta-analysis. However, in general, there is no clear evidence to support that HL intervention is effective for blood pressure management of patients with diabetes, and blood pressure may require more intensive intervention, including adjustment of drug treatment (if necessary). There was low heterogeneity ($I^{2}$ = 36.5%) among studies on self-efficacy, and the reason for this result may be that different scales measures self-efficacy. Additionally, self-efficacy was analyzed using the data of CBAs. Compared to other indicators, the lack of high-quality CBA data may affect the effect of data synthesis. Two of the data points on medication adherence came from the same study, which undoubtedly increased heterogeneity ($I^{2}$ = 88.9%). However, the results of our study have shown that medication adherence has improved, which may be due to the low level of HL of the population included in the intervention research. People with low HL had lower compliance, and the compliance of study participants were significantly improved in a short period of time after the implementation of corresponding interventions.

Study strengths: (1) The review protocol has been registered in the PROSPERO registry (CRD42021284053). (2) This systematic review has systematically and comprehensively studied the effect of HL intervention on people with diabetes. (3) To fully and accurately understand the research status, this study analyzed the distribution of countries conducting relevant intervention studies. (4) Our study involved analyzing the corresponding HL assessment tools and expounded the improvement of participants’ HL after intervention. (5) We used the GRADE approach to classify the certainty of the evidence for each outcome.

Study limitations: (1) The intervention time and follow-up time of each study were different. There were a variety of interventions and no conclusions about which were most effective. (2) Due to the lack of gray literature and the small number of included studies, publication bias cannot be completely excluded. (3) HL assessment tools were different and selected studies that focused on different levels of HL could not be effectively combined. (4) Only two studies were double-blind for both outcome measurers and participants.

## 5. Conclusions

Taken together, this systematic review and meta-analysis provided evidence that people with diabetes could benefit from HL interventions, resulting in optimized HbA1c control, increased medication adherence, enhanced self-efficacy, and an improved HL level of the participants, but the control of blood pressure was not ideal. However, as the number of studies on relevant indicators related to meta-analysis is limited, large samples are needed to further verify the reliability of results. Additionally, more rigorous high-quality intervention programs are required to assess the effect of HL.

## Data Availability

The data are not publicly available due to privacy or ethical concerns.

## Supplementary Materials

The following supporting information can be downloaded at: https://www.mdpi.com/article/10.3390/ijerph192013078/s1. Table S1: PRISMA 2020 checklist; Table S2: Search strategy; Table S3: General characteristics of included studies; Table S4: Quality assessment of included studies; Table S5: Contents of health literacy assessment tools; Table S6: GRADE summary of findings; Table S7: Sensitivity analysis of included studies.
