# Supplementary material for: The Effect of Health Literacy Intervention on Patients with Diabetes: A Systematic Review and Meta-Analysis
Source: Int J Environ Res Public Health. 2022 Oct 12;19(20):13078. doi: 10.3390/ijerph192013078 (PMC9602614; doi:10.3390/ijerph192013078)
Supplement: Supplementary file 1 [file ijerph-19-13078-s001.zip › ijerph-1909877-supplementary.pdf]

Table S1. PRISMA 2020 checklist.

Section and Topic	Item #	Checklist item	Reported on page #
**TITLE**			
Title	1	Identify the report as a systematic review.	1
**ABSTRACT**			
Abstract	2	See the PRISMA 2020 for Abstracts checklist.	1
**INTRODUCTION**			
Rationale	3	Describe the rationale for the review in the context of existing knowledge.	1-2
Objectives	4	Provide an explicit statement of the objective(s) or question(s) the review addresses.	1-2
**METHODS**			
Eligibility criteria	5	Specify the inclusion and exclusion criteria for the review and how studies were grouped for the syntheses.	2
Information sources	6	Specify all databases, registers, websites, organisations, reference lists and other sources searched or consulted to identify studies. Specify the date when each source was last searched or consulted.	2
Search strategy	7	Present the full search strategies for all databases, registers and websites, including any filters and limits used.	2
Selection process	8	Specify the methods used to decide whether a study met the inclusion criteria of the review, including how many reviewers screened each record and each report retrieved, whether they worked independently, and if applicable, details of automation tools used in the process.	2
Data collection process	9	Specify the methods used to collect data from reports, including how many reviewers collected data from each report, whether they worked independently, any processes for obtaining or confirming data from study investigators, and if applicable, details of automation tools used in the process.	2
Data items	10a	List and define all outcomes for which data were sought. Specify whether all results that were compatible with each outcome domain in each study were sought (e.g. for all measures, time points, analyses), and if not, the methods used to decide which results to collect.	3
	10b	List and define all other variables for which data were sought (e.g. participant and intervention characteristics, funding sources). Describe any assumptions made about any missing or unclear information.	3
Study risk of bias assessment	11	Specify the methods used to assess risk of bias in the included studies, including details of the tool(s) used, how many reviewers assessed each study and whether they worked independently, and if applicable, details of automation tools	2

Section and Topic	Item #	Checklist item	Reported on page #
		used in the process.	
Effect measures	12	Specify for each outcome the effect measure(s) (e.g. risk ratio, mean difference) used in the synthesis or presentation of results.	3
Synthesis methods	13a	Describe the processes used to decide which studies were eligible for each synthesis (e.g. tabulating the study intervention characteristics and comparing against the planned groups for each synthesis (item #5)).	3
	13b	Describe any methods required to prepare the data for presentation or synthesis, such as handling of missing summary statistics, or data conversions.	3
	13c	Describe any methods used to tabulate or visually display results of individual studies and syntheses.	3
	13d	Describe any methods used to synthesize results and provide a rationale for the choice(s). If meta-analysis was performed, describe the model(s), method(s) to identify the presence and extent of statistical heterogeneity, and software package(s) used.	3
	13e	Describe any methods used to explore possible causes of heterogeneity among study results (e.g. subgroup analysis, meta-regression).	/
	13f	Describe any sensitivity analyses conducted to assess robustness of the synthesized results.	3
Reporting bias assessment	14	Describe any methods used to assess risk of bias due to missing results in a synthesis (arising from reporting biases).	/
Certainty assessment	15	Describe any methods used to assess certainty (or confidence) in the body of evidence for an outcome.	3
**RESULTS**			
Study selection	16a	Describe the results of the search and selection process, from the number of records identified in the search to the number of studies included in the review, ideally using a flow diagram.	3-4
	16b	Cite studies that might appear to meet the inclusion criteria, but which were excluded, and explain why they were excluded.	3-4
Study characteristics	17	Cite each included study and present its characteristics.	4
Risk of bias in studies	18	Present assessments of risk of bias for each included study.	4
Results of individual studies	19	For all outcomes, present, for each study: (a) summary statistics for each group (where appropriate) and (b) an effect estimate and its precision (e.g. confidence/credible interval), ideally using structured tables or plots.	4-6

Section and Topic	Item #	Checklist item	Reported on page #
Results of syntheses	20a	For each synthesis, briefly summarise the characteristics and risk of bias among contributing studies.	4-6
	20b	Present results of all statistical syntheses conducted. If meta-analysis was done, present for each the summary estimate and its precision (e.g. confidence/credible interval) and measures of statistical heterogeneity. If comparing groups, describe the direction of the effect.	5-6
	20c	Present results of all investigations of possible causes of heterogeneity among study results.	5-6
	20d	Present results of all sensitivity analyses conducted to assess the robustness of the synthesized results.	5-6
Reporting biases	21	Present assessments of risk of bias due to missing results (arising from reporting biases) for each synthesis assessed.	/
Certainty of evidence	22	Present assessments of certainty (or confidence) in the body of evidence for each outcome assessed.	5-6
**DISCUSSION**			
Discussion	23a	Provide a general interpretation of the results in the context of other evidence.	6-8
	23b	Discuss any limitations of the evidence included in the review.	6-8
	23c	Discuss any limitations of the review processes used.	6-8
	23d	Discuss implications of the results for practice, policy, and future research.	6-8
**OTHER INFORMATION**			
Registration and protocol	24a	Provide registration information for the review, including register name and registration number, or state that the review was not registered.	2
	24b	Indicate where the review protocol can be accessed, or state that a protocol was not prepared.	2
	24c	Describe and explain any amendments to information provided at registration or in the protocol.	2
Support	25	Describe sources of financial or non-financial support for the review, and the role of the funders or sponsors in the review.	8
Competing interests	26	Declare any competing interests of review authors.	8
Availability of data, code and other materials	27	Report which of the following are publicly available and where they can be found: template data collection forms; data extracted from included studies; data used for all analyses; analytic code; any other materials used in the review.	8

Table S2. Search strategy.

SCIE	
#1	TS = (diabetes mellitus OR dm OR diabetes OR diabetic mellitus OR diabetic OR mellitus)
#2	TS = health literacy
#3	#1 AND #2
PubMed	
#1	(diabetes mellitus [Title/Abstract]) OR (dm [Title/Abstract]) OR (diabetes [Title/Abstract]) OR (diabetic mellitus [Title/Abstract]) OR (diabetic [Title/Abstract]) OR (mellitus [Title/Abstract])
#2	health literacy [Title/Abstract]
#3	#1 AND #2
Embase	
#1	"diabetes mellitus": ti, ab OR "dm": ti, ab OR "diabetes": ti, ab OR "diabetic mellitus": ti, ab OR "diabetic": ti, ab OR "mellitus": ti, ab
#2	health literacy: ti, ab
#3	#1 AND #2

* All searches were conducted in October 10, 2021.

Table S3. General characteristics of included studies.

Author, year	Study type	Sample size		HL score		Measure		Intervention time	Geographic area
		Intervention	Control	Intervention Mean (SD)	Control Mean (SD)	Intervention	Control		
Whittemore R, 2020	RCT	26	21	——	——	custom education + self-management + text message program (7 interactive group-based educational sessions on diabetes self-management; text message bank consisted of 181 messages, approximately 20-30 messages for each process of behavior change; 7 weekly sessions and 6 moth of daily text/picture messages)	custom education	6months	Mexico
Zeidi IM, 2021	RCT	83	83	93.27(12.72)	92.86(11.89)	five 45-minute training sessions (each session was used such asecture, role playing, focus group discussion, Q&A methods, brain storming and practical implementation of skills.); 10-minute educational film	custom education	Not mentioned	Iran
Seidling HM, 2020	RCT	55(29)	58(44)	——	——	electronic medication module intervention (access to an internet-based medication module allowing them to store their medication information, look up drug information, and print a medication schedule)	information brochure education (received a brochure about the importance and content of a medication schedule)	4-8weeks	Germany
Sugita H,2017	RCT	21(20)	20(19)	2.90(0.32)	3.05(0.45)	text message-based HL intervention (created by medical professionals, including physicians and a clinical psychotherapist, on the basis of the HL scales)	medication reminder text message ("please do not forget to take your medication")	6 months	Japan

Ghisi GLM,2020	CBA	84(47)	——	METER: 34.73(6.42) NVS: 4.00(1.91)	——	evidence- and theoretically-based comprehensive education intervention (offered supervised classes once a week for 24 weeks, and provided a home exercise prescription for the other 6 days of the week)	——	6 months	Canada
Long AF,2012	CBA	319(156)	——	——	——	PACCTS intervention (focus lay on increasing patient knowledge and understanding of diabetes and individual self-management elements, such as monitor HbA1c, diet, exercise and medication adherence, and then onto a focus on more general self-care to enable)	——	2 years	England
Dang YH,2020	RCT	13(11)	12(12)	REALM-R: 4.1 (2.2) DNT-5: 1.3 (1.3)	REALM-R: 2 (2.5) DNT-5: 1.3 (1.8)	literacy-appropriate intervention (received the teach-back method and action plans at every clinic visit to re-assess their knowledge; follow-up phone calls were conducted every 2-4 weeks)	medication management and diabetes education	3 months	US
Wei Y,2020	RCT	200(160)	200（167）	HeLMS: 116(104,120) [a] C-DNT-5: 80 (60,100) [a]	HeLMS: 116(113,120) [a] C-DNT-5: 100 (80,100) [a]	HL intervention (the PRIDE and a Clear Health Communication Curriculum)	custom education (conventional clinical consultations, and treatment provided based on existing knowledge and at the individual clinician's discretion)	1 year	China
Kim MT,2020	RCT	120(105)	130(104)	REALM: 34.2 (2.1) DM-REALM: 54.2 (2.1) TOFHLA: 4.3(0.3) NVS: 1.7(0.2)	REALM: 30.0(2.1) DM-REALM: 48.4 (2.5) TOFHLA: 4.1(0.4) NVS: 1.7(0.2)	HL-focused intervention (weekly 2-hour didactic classes for 6 weeks, classes focused on DM etiology, treatment regimens, and training focused on HL and communication with healthcare providers; monthly telephone counseling; home	custom education	12 months	US

						monitoring of daily blood sugar)			
Han HR,2019	CBA	19(11)	——	LAD: 59.5(0.9) NVS: 1.7(1.2)	——	health literacy enhanced intervention (consisted of 4-week health literacy training and disease knowledge education followed by two home visits and monthly phone counseling for over 24 weeks)	——	28 weeks	US
Calderón JL,2014	RCT	118(113)	122(109)	——	——	animation intervention (Spanish/ English 13-minute animated video; video conveys content from 3 main areas: general information about diabetes, clinical management, and self-management)	text intervention (provided with easy-to-read diabetes information available from the National Diabetes Information Clearinghouse of the National Institute of Diabetes and Digestive and Kidney Diseases (NIDDK))	Not mentioned	US
Moura NDS,2019	QEs	55	——	——	——	educational intervention (comprised three meetings that sought to train type 2 DM patients for the adoption of appropriate behaviors considering their condition, each meeting lasted approximately 60 minutes)	——	Not mentioned	Brasil
Hung JY,2017	QEs	49	46	63.3(5.4)	67.1(4.6)	DCMP-based group education intervention (received 7 DCMP-based group education sessions (10–12 participants, 1.5 hours each) for 7 consecutive weeks in addition to routine health education at a private room in the outpatient unit)	custom education (usual health education lasting for about 20 minutes after per medical visit which consisted of consultation from ward nurses on disease symptoms, related treatment options, and clarification of doctors' orders)	2 months	Taiwan
Negarandeh R,2013	RCT	i： 45(44) ii： 45(43)	45(40)	i: 34.84(15.70) ii:	33.58(16.46)	i： pictorial image educational strategy intervention (received the education via illustrated	custom education (present an educational brochure containing information	3 weeks	Iran

				34.71(14.60)		contents within three weekly sessions, each lasting 20 min)<br>ii : teach back educational strategy intervention (received the education based on teach back strategy within three weekly sessions, each lasting 20 min)	regarding diabetes control and to answer to patients' questions by the same community health nurse in a similar time to the intervention groups)		
Gharachourlo M,2018	RCT	50(42)	50(42)	9.95(2.52)	10.36(2.14)	custom education + health literacy intervention (attended six sessions of counselling with a health literacy approach in addition to counselling on routine pregnancy care)	custom education (attended counselling sessions on safe pregnancy care and received a training package)	6 weeks	Iran
McGowan P,2019	CBA	115	——	HL-read:<br>1.60 (1.12)<br>HL-learning:<br>4.39 (1.02)<br>HL-fill out forms:<br>1.60 (1.12)	——	low-cost telephone peer-coaching intervention (consisted of weekly 30-min telephone calls by coaches to patients for a period of 6 months)	——	6 months	Canada
Kim MT,2015	RCT	120(105)	130(104)	——	——	SHIP-DM intervention (consisted of key self-management skill-building activities through 12 hours of group education sessions, followed by integrated counseling and behavioral coaching by a team of RNs and community health workers)	delayed intervention (received a brief educational brochure at baseline that highlighted the critical self-management principles of SHIP-DM)	12 months	US
Prabsangob K,2019	QEs	35	35	36.40 (8.98)	38.40(9.69)	SHG intervention (health promotion program was implemented by using SHG techniques)	custom education	3 months	Thailand
Swavely D,2014	CBA	277(106)	——	Adequate: 67 (63.2)<br>Marginal:	——	LHL diabetes education (consisted of both individualized and group	——	12 months	US

				12 (11.3) Inadequate: 27 (25.5)		diabetes education in English and Spanish languages for adults with type 2 diabetes, encompassing 13 hours of education over 12 weeks)			
Lachance L,2018	CBA	161	——	——	——	NKFM intervention programs (Evidence-based chronic disease prevention and management interventions; Health literacy mentoring interventions)	——	Not mentioned	US
Wolf MS,2014	QEs	214	272	28.5 (Limited HL, %)	34.6 (Limited HL, %)	clinic-based health literacy intervention (involved practice re-design to routinely provide brief diabetes education and counseling services, set action-plans, and perform follow-up without additional financial resources)	outsourced implementation health literacy intervention (clinics referred patients to a telephone-based diabetes educator for the same services)	Not mentioned	US

*SD: standard difference; RCT: randomized controlled trials; CBA: controlled before-and-after trials; METER: the Medical Term Recognition Test; NVS: the Newest Vital Sign; REALM-R: Rapid Estimate of Adult Literacy in Medicine–Revised; DNT-5: 5-item version of the Diabetes Numeracy Test; HeLMS: the Health Literacy Management Scale (Chinese versions); C-DNT-5: 5-item Chinese version of the Diabetes Numeracy Test; a: median (interquartile range); REALM: ; DM-REALM: ; TOFHLA: ; LAD: ; HL: health literacy; QEs: quasi-experimental studies; Q&A: Questions & Answers; PACCTS: proactive, call centre treatment support; PRIDE: partnership to improve diabetes education toolkit; DCMP: diabetes conversation map program; SHIP-DM: a community-based self-help intervention program for diabetes; SHG: self-help group; LHL: low health literacy; NKFM: national kidney foundation of Michigan; US: the United States.

Table S4. Quality assessment of included studies.

Author,year	Type	①	②	③	④	⑤	⑥	⑦	⑧	⑨	Score
Whittemore R, 2020	RCT	Yes	Yes	Yes	Yes	Yes	Yes	Unclear	Yes	Yes	8
Zeidi IM, 2021	RCT	Yes	Unclear	Yes	Yes	Yes	Unclear	Unclear	Yes	Yes	6
Seidling HM, 2020	RCT	Yes	No	Yes	Yes	Yes	No	Unclear	Yes	Yes	6
Sugita H,2017	RCT	Yes	Unclear	Yes	Yes	Yes	Unclear	Unclear	Yes	Yes	6
Ghisi GLM,2020	CBA	No	No	Yes	Yes	Yes	Unclear	Unclear	Yes	Yes	5
Long AF,2012	CBA	No	No	Yes	Yes	Yes	Unclear	Unclear	Yes	Yes	5
Dang YH,2020	RCT	Unclear	Unclear	Yes	Yes	Yes	Unclear	Unclear	Yes	Yes	5
Wei Y,2020	RCT	Unclear	Unclear	Yes	Yes	Yes	Unclear	Unclear	Yes	Yes	5
Kim MT,2020	RCT	Unclear	Unclear	Yes	Yes	Yes	Unclear	Unclear	Yes	Yes	5
Han HR,2019	CBA	No	No	Yes	Yes	Yes	Unclear	Unclear	Yes	Yes	5
Calderón JL,2014	RCT	Yes	Yes	Yes	Yes	Yes	Yes	Unclear	Yes	Yes	8
Moura NDS,2019	QEs	No	No	Yes	Yes	Yes	Unclear	Unclear	Yes	Yes	5
Hung JY,2017	QEs	No	No	Yes	No	Yes	Unclear	Unclear	Yes	Yes	4
Negarandeh R,2013	RCT	Yes	Yes	Yes	Yes	Yes	Unclear	Unclear	Yes	Yes	7
Gharachourlo M,2018	RCT	Unclear	Unclear	Yes	Yes	Yes	Unclear	Unclear	Yes	Yes	5
McGowan P,2019	CBA	No	No	Yes	Yes	Yes	Unclear	Unclear	Yes	Yes	5
Kim MT,2017	RCT	Unclear	Unclear	Yes	Yes	Yes	Unclear	Unclear	Yes	Yes	5
Prabsangob K,2019	QEs	No	No	Yes	Yes	Yes	Unclear	Unclear	Yes	Yes	5
Swavely D,2014	CBA	No	No	Yes	Yes	Yes	Unclear	Unclear	Yes	Yes	5
Lachance L,2018	CBA	No	No	Yes	Yes	Yes	Unclear	Unclear	Yes	Yes	5
Wolf MS,2014	QEs	No	Unclear	Yes	No	Unclear	Unclear	Unclear	Yes	Yes	3

* RCT: randomized controlled trials; CBA: controlled before-and-after trials; QEs: quasi-experimental studies; ①: random sequence generation; ②: allocation concealment; ③: baseline outcome measurements similar; ④: baseline characteristics similar; ⑤: incomplete outcome data; ⑥: knowledge of the allocated interventions adequately prevented during the study; ⑦: protection against contamination; ⑧: selective outcome reporting; ⑨: other risks of bias.

Table S5. Contents of health literacy assessment tools.

Health literacy assessment tool	Scoring	Measurement properties	Content
TOFHLA	Scores range 0-100: <60 = inadequate HL, 60-75=marginal HL, >75=adequate HL	Reliability: Cronbach's alpha: 0.98; Validity: 0.84 (with REALM), 0.74 (with WRAT-R)	① reading comprehension; ② numerical ability
s-TOFHLA	Scores range 0-36: 0-16 = inadequate HL, 17-22 = marginal HL, 23-36 = adequate HL	Reliability: Cronbach's alpha: 0.98; Validity: 0.91 (with TOFHLA), 0.80 (with REALM)	① reading comprehension; ② numerical ability
NVS	Scores range1-6, score <4 = limited HL	Reliability: Cronbach's alpha: 0.76; Validity: 0.59 (with TOFHLA), AUROC: 0.88 (based on TOFHLA), Screening sensitivity: 0.72 (based on TOFHLA), Screening specificity: 0.87 (based on TOFHLA)	① reading skill; ② numeracy skill; ③ comprehension skill
REALM	Scores range 0-66: 0-18=≤ 3rd grade, 19-44=4th-6th grade, 45-60=7th-8th grade, 61-66 = ≥9th grade	Reliability: Cronbach's alpha: 0.99, ICC: 0.92; Validity: 0.97 (with PIAT-R), 0.96 (with WRAT-R), 0.88 (with SORT-R)	① reading skill; ② interpretation skill
REALM-R	Scores range 0-8, Score ≤6=poor HL	Reliability: Cronbach's alpha: 0.91; Validity: 0.72 (with REALM), 0.64 (with WRAT-R)	① reading skill; ② interpretation skill
FCCHL	Scores range 1-4, higher scores indicate higher HL level	Reliability: Cronbach's alpha: overall scale: 0.78, Functional domain: 0.84, Communicative domain: 0.77, Critical domain: 0.65	① functional; ②communicative; ③critical
METER	1=correct answer, 0=incorrect answer;	Reliability: Cronbach's alpha: 0.93; Validity: 0.74 (with REALM)	① vocabulary knowledge; ② reading comprehension; ③ verbal fluency; ④ cultural literacy
SILS	Possible scores are: 0 (never), 1 (rarely), 2 (sometimes), 3 (often), 4 (always); Score>2 = difficulty with reading printed health material	AUROC: 0.64-0.87 (based on s-TOFHLA), Screening sensitivity: 0.47-0.73 (based on s-TOFHLA), Screening specificity: 0.83 (based on s-TOFHLA)	①reading skill
Health Literacy Questionnaire (HLQ)	Scoring: 4-point scale(ranging from 1 = strongly disagree to 4 = strongly agree)+5-point scale (from 1 = cannot do or usually difficult to 5 = very easy); higher scores indicating potential HL strengths	Reliability: Cronbach's alpha: >0.77	① feeling understood and supported by healthcare providers; ② having sufficient information to manage my health; ③actively managing my health; ④ social support for health; ⑤appraisal of health information; ⑥ ability to actively engage with healthcare providers; ⑦navigating the healthcare system; ⑧ability to find good health information; ⑨ understanding health information well enough to know what to do

DM-REALM	Scores range 0-82, 1=correct answer, 0=incorrect answer	Reliability: Cronbach's alpha: 0.98, Validity: 0.49 (with FHL)	① reading skill
LAD	Scores range 0-60, 0-20 = ≤4th grade, 21-40 = 5th-9th grade, 41-60 = ≥9th grade	Reliability: ICC: 0.86; Validity: 0.90 (with REALM), 0.81 (with WRAT-3)	① literacy skill
HeLMS (Chinese version)	Scores range 28-140, 1 (completely unable or completely unwilling), 2 (very difficult or very reluctant), 3 (a certain degree of difficulty and a moderate degree of willingness), 4 (little difficulty and more willingness), 5 (no difficulty or great willingness)	Reliability: Cronbach's alpha: 0.894, Validity: 0.683	① ability to access information; ② ability to communicate and interact; ③ willingness to improve health; ④ willingness to provide financial support
C-DNT-5	Items were scored as correct or incorrect, and scores were reported as the percent correct with a possible range from 0% to 100%	————————	① counting nutrients; ② physical activity; ③ blood glucose monitoring; ④ oral dose adjustment; ⑤dose capacity of insulin injection
DNT-5	Items were scored as correct or incorrect, and scores were reported as the percent correct with a possible range from 0% to 100%	————————	① counting nutrients; ② physical activity; ③ blood glucose monitoring; ④ oral dose adjustment; ⑤dose capacity of insulin injection
DHLS	————————	Reliability: coefficient alpha =0.79	① general T2D information; ② clinical management information; ③self-management; ④ ethnomedical (cultural) beliefs.
DM health literacy questionnaire	Scores range 0-100, total scores indicating more positive diabetic HL	Reliability: Cronbach's alpha: 0.90; Validity: >0.80	————————
IHLQ	Scores range 0-113	Reliability: Cronbach's alpha: 0.71~0.96; Validity: Kaiser-Meyer-Olkin (KMO) :0.95, Bartlett's test result of 3.017 with P < 0.001	① ability to access health information; ② health information use; ③ reading skills; ④ comprehension skills; ⑤ assessment and judgment skills; ⑥ decision-making and communication skills; ⑦health knowledge; ⑧ individual empowerment; ⑨ social empowerment
3-SQ	Scores range 0-12, high scores = high HL skills, low scores = low HL skills	AUROC: 0.66-0.87 (based on s-TOFHLA), 0.72-0.84 (based on REALM); screening sensitivity:0.48-0.60 (based on s-TOFHLA), screening specificity: 0.79-0.83 (based on s-TOFHLA)	① help read; ② problems reading; ③ confident with forms

* TOFHLA: the Test of Functional Health Literacy in Adults; s-TOFHLA: Test of Functional Health Literacy in Adults–Short Form; NVS: the Newest Vital Sign; REALM: the Rapid Estimate of Adult Literacy in Medicine; REALM-R: Rapid Estimate of Adult Literacy in Medicine–Revised; FCCHL: Functional, Communicative, and Critical Health Literacy Scale; METER: the Medical Term Recognition Test; SILS: Single Item Literacy Screener; HLQ: Health Literacy Questionnaire; DM-REALM: Diabetes-Focused Print Health Literacy Scale Using the Rapid Estimate of Adult Literacy in Medicine; LAD: the Literacy Assessment in Diabetes; HeLMS: the Health Literacy Management Scale (Chinese

version); C-DNT-5: 5-item Chinese version of the Diabetes Numeracy Test; DNT-5: 5-item version of the Diabetes Numeracy Test; DHLS: Diabetes Health Literacy Survey; DM: diabetes mellitus; IHLQ: Iranian Health Literacy Questionnaire; 3-SQ: 3-item version of Screening Questions; HL: health literacy; WRAT-R: Wide Range Achievement Test-Revised; AUROC: receiver operating characteristic curve; ICC: intraclass correlation coefficient; PIAT-R: Peabody Individual Achievement Test-Revised; SORT-R: Slosson Oral Reading Test-Revised; T2D: Type 2 diabetes.

Table S6. GRADE summary of findings.

Certainty assessment							№ of patients		Effect	Certainty	Importance
№ of studies	Study design	Risk of bias	Inconsistency	Indirectness	Imprecision	Other considerations	health literacy intervention	usual care	Absolute (95% CI)		
**HbA1c**											
4	randomised trials	not serious	not serious	not serious	serious[a]	none	354(296)	362(302)	**WMD** =-0.44, **95%CI** (-0.64 to -0.24)	⊕⊕⊕◯ Moderate	CRITICAL
**SBP**											
2	randomised trials	not serious	not serious	not serious	serious[a]	none	320(265)	330(271)	**SMD** =-0.13, **95%CI** (-0.3 to 0.04)	⊕⊕⊕◯ Moderate	IMPORTANT
**Self-efficacy**											
3	observational studies (before-after studies)	serious[b]	not serious	not serious	not serious	none	441(232)	-----	**SMD** =0.85, **95%CI** (0.66 to 1.05)	⊕◯◯◯ Very low	IMPORTANT
**Medication adherence**											
3	randomised trials	not serious	serious[c]	not serious	serious[d]	none	111(107)	110(104)	**SMD**= 1.32, **95%CI** (0.37 to 2.28)	⊕⊕◯◯ Low	IMPORTANT

* HbA1c: glycosylated hemoglobin; SBP: systolic blood pressure; CI: confidence interval; SMD: standardized mean difference; WMD: weighted mean difference.

Explanations

a. Serious imprecision for less than 400 participants.

b. The three studies were controlled before-and-after trials, which could not be randomized and it was difficult to achieve double-blind of the outcome measurer and the research participant. The loss to follow-up rate of two studies was more than 20%.

c. I-squared=88.9%, P=0.000.

d. Serious imprecision for less than 400 participants and the confidence interval was wide.

Table S7. Sensitivity analysis of included studies.

Sensitivity analysis	Random effect model	Fixed effect model	Stability
HbA1c	WMD= -0.78, 95%CI (-0.94, -0.62), P=0.00	WMD= -0.78, 95%CI (-0.94, -0.62), P=0.00	Yes
SBP	SMD= -0.05, 95%CI (-0.34, 0.25), P=0.75	SMD= -0.04, 95%CI (-0.20, 0.12), P=0.64	Yes
Self-Efficacy	SMD= 0.83, 95%CI (0.55, 1.10), P=0.00	SMD= 0.85, 95%CI (0.65, 1.04), P=0.00	Yes
Medication Adherence	SMD= 1.85, 95%CI (0.19, 3.52), P=0.03	SMD= 1.89, 95%CI (1.54, 2.23), P=0.00	Yes

* HbA1c: glycosylated hemoglobin; SBP: systolic blood pressure; CI: confidence interval; SMD: standardized mean difference; WMD: weighted mean difference.
